# Recovery for Professional and Elite Amateur Golfers: A Scoping Review of Evidence-Based Methods

**DOI:** 10.1007/s40279-025-02297-0

**Published:** 2025-08-21

**Authors:** Chris Bishop, Jiaqing Xu, Laura Wilson, Graeme Close, Margo Mountjoy, David Dunne, Daniel Coughlan, Anthony Turner, Andrew Murray

**Affiliations:** 1 London Sport Institute, Faculty of Science and Technology, Middlesex University, The Burroughs, London, NW4 4BT UK; 2 The R&A, St. Andrews, Scotland, UK; 3 PGA European Tour Health and Performance Institute, Virginia Water, Surrey, UK; 4 Ladies European Tour, Buckinghamshire Golf Club, Buckingham, UK; 5 Research Institute for Sport and Exercise Sciences, Liverpool John Moores University, Liverpool, UK; 6 Department of Family Medicine, McMaster University, Hamilton, ON Canada; 7 International Golf Federation, Lausanne, Switzerland; 8 Hexis Nutrition, Dublin, Ireland; 9 England Golf, Woodhall Spa, Lincolnshire, UK

## Abstract

The lifestyle and athletic demands of a professional or elite amateur golfer are both physically and mentally challenging. Players need to withstand large forces during the swing, frequently travel between time zones, and often cycle through a variety of training and competition environments for large portions of the competitive season. Thus, with numerous factors contributing to physical and cognitive stress, optimising recovery for golfers is paramount. The primary objective of this scoping review was to evaluate different evidence-based recovery methods for professional and elite amateur golfers and assess where the current research gaps lie. A three-step search strategy identified relevant primary and secondary articles, in addition to the grey literature, using a total of five online databases (SPORTDiscus, Scopus, Web of Science, ProQuest Central and PubMed), which retrieved articles from January 2000 to May 2024. Data were extracted using a standardised tool to create a descriptive analysis and a thematic summary. Studies were included if they focused on nutritional and hydration methods, laboratory and controlled environment methods, sleep and jet leg management, independent methods or adjunct recovery methods, in relation to golf or other sporting populations. The initial search found 4862 relevant articles from the selected databases, with 39 studies meeting our criteria for the scoping review. Limited investigations have been conducted examining effective recovery methods for golfers. However, some preliminary evidence supports the use of targeted nutrition and hydration strategies, massage, and regular mobility and flexibility exercise. In addition, though, a more fundamental focus on sleep and jet lag management strategies is required, given the lifestyle challenges often faced by professional and elite amateur players. If golfers want to improve their chances of consistently competing at the highest level, strategies that focus on optimising recovery for superior health and well-being are essential for helping to sustain performance over time.

## Key Points

**Table:** 

Professional and elite amateur golfers often travel and live in different time zones for brief periods of time, owing to the demands of competitive golf.
Further to this, the golf swing itself may generate forces up to eight times a player’s body mass, with approximately 2000 swings performed in a given tournament week, resulting in a large volume of cumulative stress on the body. Thus, recovery for golfers is critical in order to optimise preparation for tournaments.
Empirical research pertaining to recovery methods for golfers is scarce, although some preliminary evidence points towards the benefit of targeted nutrition and hydration strategies, massage, and regular mobility and flexibility exercise.
Beyond these and of particular importance, sleep and nutrition should be considered cornerstones in any player’s over-arching preparation given the travelling demands associated with the sport at the professional and elite amateur level.

## Introduction

There are a number of professional golf tours around the world, with the most prominent ones being the Professional Golfers Association (PGA) Tour, DP World Tour and LIV golf in the male game, and the Ladies PGA Tour (LPGA) and Ladies European Tour (LET) in the female game. It is important to note that some players compete on multiple professional tours. Some of these professional tours run for the majority of the year, with no clear off-season, unless a golfer chooses to take a period of time where they do not compete [1]. For example, in 2024, the DP World Tour hosted 44 events in 24 countries that spanned across five continents. With the exception of events on the LIV golf tour, most professional tournaments take place over 4 days from Thursday to Sunday. However, if golfers perform poorly (i.e. below average relative to those players who are competing in that competition), they will fail to ‘make the cut’ for Saturday and Sunday. Further to this, it is common practice for players to arrive for a tournament on the Monday or Tuesday, in order to start their pre-tournament preparations [2], which may include recovery from previous competition and travel, physical preparation, working on particular technical aspects of their game, acclimatising to the new location and familiarising themselves with the golf course.

Consequently, the performance lifestyle of a professional golfer is highly variable. Tournaments are in a different location every week, with many professional tours showcasing a wide geographical dispersion for events. Thus, regular travel and changing time zones are a routine part of a golfer’s lifestyle, coupled with 'living out of a suitcase' for extended periods of time, often staying in hotels and house sharing with members of their inter-disciplinary team (e.g. swing coach, strength and conditioning coach, physical therapist) [2]. Naturally, these factors in a golfer’s lifestyle may have a series of 'knock-on' effects on their over-arching health, well-being and performance, such as inconsistent (i) sleep patterns, (ii) nutrition and hydration provision, (iii) physical and cognitive training regimes, and (iv) access to equipment, facilities and personnel, to name a few. Consequently, and also when considered cumulatively, this may lead to a reduction in performance on the golf course. Although this is challenging to quantify, it is intuitive that consistently gaining enough sleep and relaxation time, making sensible choices around nutrition and hydration, and having a consistent high-quality fitness regime are beneficial for any athlete’s health, wellness and sporting performance [1, 3].

Beyond these lifestyle factors, practitioners working with professional golfers must also have a comprehensive understanding of the biomechanical demands imposed on the body during the golf swing. For example, previous literature has suggested that compressive forces on the spine exceed 7000 Newtons (N), when swinging a driver, wood or long iron [4]. Whilst there are a wide range of body types in professional golf, such forces would exceed eight times the body mass in a single swing, for a player weighing 80 kg. This is supported by Dale and Brumitt [5], who showed that peak compression forces during a full back swing were 7.6 ± 0.4 times body mass, and still 7.0 ± 0.5 times body mass with a shortened back swing. Further to this, it has also been suggested that during a single tournament week, professional players may perform over 2000 swings when competition and practice time are combined [6]. Further still, with each 18-hole round being 6.4–11.3 km in distance [7], players may cover distances of 40–80 km of golf-related walking, per week. Thus, travel demands, prolonged concentration during training and competition, and pressure during competition itself are also cognitively demanding [8]. Consequently, the physical and likely psychological demands of professional golf are substantial and likely to be greater than many realise. Thus, players may be able to improve their ability to tolerate these aforementioned demands by physiological and/or psychological improvements attained from appropriately applied recovery strategies.

With this in mind, a range of methods can be utilised for athletes to enhance and optimise recovery. For example, one of the simplest and most effective recovery methods is adequate quality and duration of sleep, with previous research indicating that persistent sleep disturbance can have significant negative effects on athlete performance [9]. For professional golfers, the week-to-week time zone adjustments represent a considerable challenge in terms of retaining a routine sleep pattern. Additionally, good-quality nutrition and hydration are essential to ensure that the body has adequate energy availability, protein for muscle synthesis and repair capabilities following athletic tasks [10], and macro and micronutrients to assist the immune system [11]. Finally, other methods of recovery may also add value, such as (i) manual therapy, (ii) foam rolling, (iii) ice baths, and (iv) general warm-up and cool-down routines [12]. However, golf is not a sport with a long-standing history of utilising physical and mental preparation and recovery to complement player health and performance [13, 14]. Furthermore, with a growing body of literature now outlining the advantages of being able to hit the ball further [13, 15–19], professional players are taking physical preparation strategies more seriously, pushing their physical limits in an attempt to gain a competitive advantage over other players. Collectively, the aforementioned information provides a strong rationale for practitioners to better understand the most effective methods to enhance the recovery process for professional golfers, and serves as the primary aim of this scoping review.

## Methods

This review was conducted in accordance with the Preferred Reporting Items for Systematic Reviews and Meta-Analyses extension for Scoping Reviews (PRISMA-ScR) [20] and the well-established ‘five stages’ as suggested by Arksey and O’Malley [21], incorporating suggestions from Levac et al. [22] and Peters et al. [23]. The following information summarises the approach of each stage during the scoping review process.

### Stage 1: Identification of the Research Question

Considering the study population (i.e. professional and elite amateur golfers) and the aforementioned lifestyle challenges they are exposed to, this enabled a broad research question to be formulated: *“What are the most effective recovery modalities or strategies for professional or elite amateur golfers to enhance the recovery process?”.*

### Stage 2: Identification of the Relevant Literature

The following inclusion and exclusion criteria were developed through researcher discussion between the author team and wider expert consultation (consisting of industry experts).

#### Inclusion Criteria

- All healthy age groups across all genders.
- All types of literature evaluating recovery strategies amongst professional and elite amateur golfers (source type including: primary and secondary empirical studies, review studies, case studies, guidelines, as well as the grey literature to include unpublished and ongoing trials, annual reports, dissertations and conference proceedings).
- All manuscripts written in English.
- Full texts available online.

#### Exclusion Criteria

- Studies investigating the effects of post-injury recovery strategies.
- Opinion pieces (e.g. magazines, newspaper articles, papers or reports with no data, trade journals, wire feeds).

#### Search Strategies and Databases

The search strategy was designed to discover both the published and unpublished literature. An initial limited search of ProQuest Central and Google Scholar was conducted to identify relevant articles on the topic. The text words contained in the titles and abstracts of applicable articles, and the index terms used to describe the articles, were used to develop a full search strategy for SPORTDiscus, Scopus, Web of Science, ProQuest Central and PubMed electronic databases (publication date from January 2000 to May 2024). The reference list of the included articles was screened for additional studies, while additional searches were conducted in Google Scholar and ResearchGate™. Two researchers (CB and JX) performed the searches independently. The descriptors used were “golf”, “golfers”, “recovery”, “recovery strategies”, “regeneration” and “replenish”. The final structure of words with the descriptors and Boolean operators together was ([golf, athletes, athlete] AND [recovery strategies] OR [sleep, downtime] OR [nutrition, hydration] OR [jet lag, travel] OR [compression, ice baths, infra-red]). The search strategy, including all identified keywords, index terms and filters, was adapted for each aforementioned database to execute some of the exclusion criteria and avoid an excess of articles unrelated to our intended search. Finally, the reference lists of all the included literature were evaluated for any additional relevant sources of information.

### Stage 3: Study Selection

**Fig. 1:**
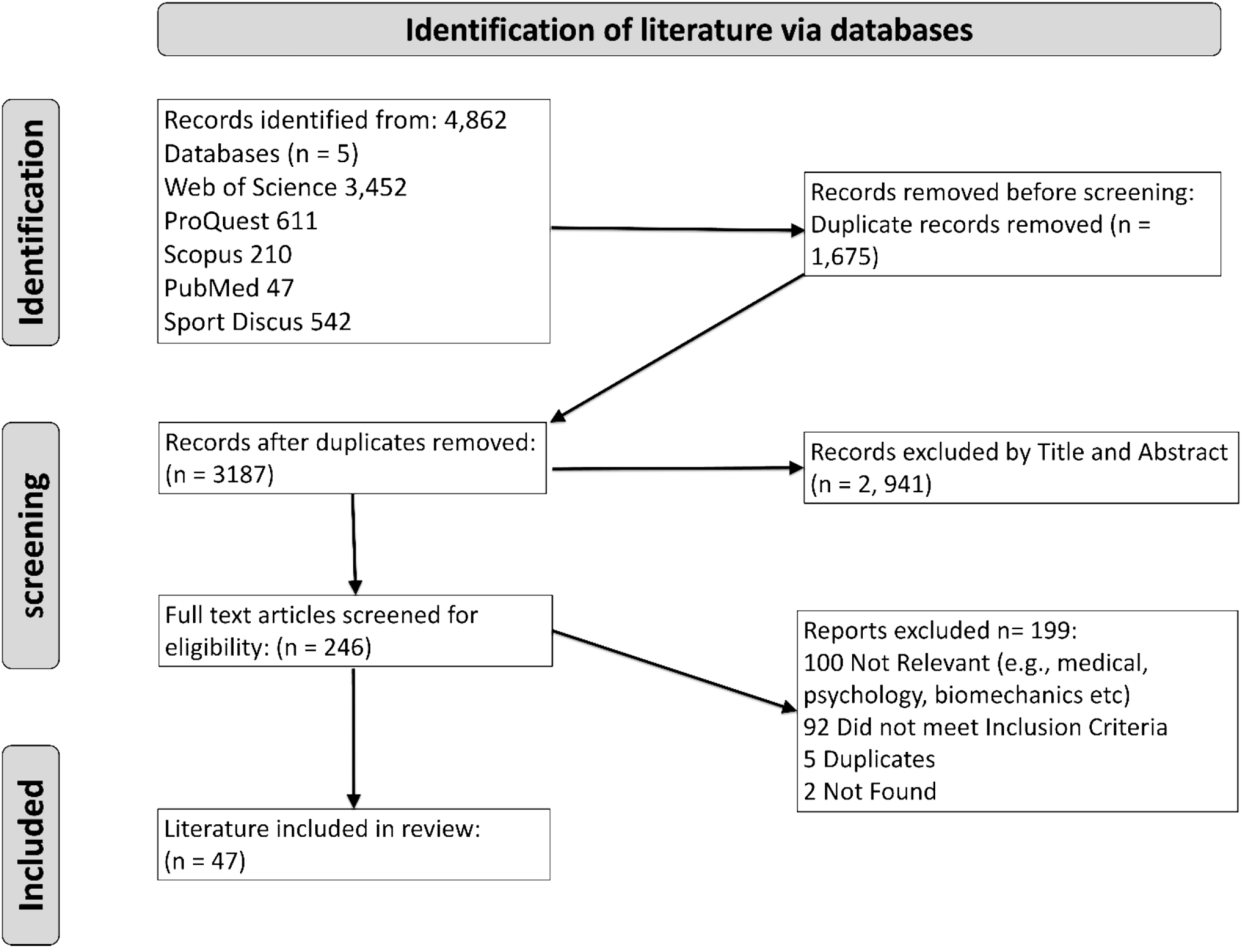
Scoping review flow chart

Following the search, all the identified literature was collated and exported to an Excel spreadsheet. Two researchers (CB and JX) independently screened the titles and abstracts and removed literature according to the inclusion and exclusion criteria. The remaining body of literature was then read in full, and the decision of whether to include it was made between two researchers (CB and JX). In the event of any disagreements, a third researcher was consulted (AM) to discuss and reach a final decision. The details of the search and exclusion reasons are reported in Fig. 1.

### Stage 4: Charting the Data

The charting of tables to record and extract the variables of interest from the included body of literature was developed by two researchers (CB and JX), with the process being initiated by one author (JX) and verified by a second (CB). If any discrepancies arose, a third reviewer (AM) was consulted to settle the disagreement. The data charting process was carried out in Microsoft Excel by fully reading all of the included literature and transferring the variables of interest to the form. In the instance of any missing or relevant information, authors were contacted directly via e-mail, in an attempt to answer queries. Data extraction categories included:

A. Lead author.
B. Year of publication.
C. Publication format (journal, conference, thesis).
D. Aims and/or purpose.
E. Study population and sample size (e.g. sex, handicap, years’ experience, competitive level).
F. Recovery strategy characteristics (e.g. intervention type).
G. Duration of strategy.
H. Outcomes and details (e.g. how to measure outcomes, benchmark of outcome measures).
I. Key findings pertaining to the aim of the present scoping review.

### Stage 5: Collating, Summarising and Reporting the Results

The methods employed in this scoping review were in accordance with the protocol suggested by Murray and colleagues [24], which enabled us to collate existing knowledge on this topic and report as:

- A descriptive analysis, mapping the data, showing distribution of the literature by publication period, country of origin, study method and theme/focus.
- A thematic analysis, characterising how the identified literature related to the research question and aims, and the main findings from these organised by theme.

Additionally, we aimed to:

- Map the evidence and key concepts available for golf and recovery strategies.
- Report and summarise existing research findings for golfers, support staff practitioners (e.g. swing coaches, physiotherapists, sport scientists, strength and conditioning coaches) and any other relevant stakeholders.
- Identify gaps in the existing literature on recovery methods in golf, in an attempt to help guide future empirical investigations in the sport.

## Results and Discussion

### Descriptive Analysis

A (PRISMA-ScR) flow diagram was produced to report the results from the search and literature selection process (Fig. 1). The initial search found 4862 relevant articles from the selected databases, and 3187 papers remained after removing duplicate records. Once records were excluded by their title and abstract, a further 2941 records were removed, and 246 articles then remained. A further seven papers were excluded because of being duplicates and full texts not being available, resulting in 246 papers meriting a full-text review. This present scoping review identified 47 eligible articles that were relevant to the aim and research question: *“What are the most effective recovery modalities or strategies for professional or elite amateur golfers to enhance the recovery process?”.*

### Characteristics of Studies

#### Geography of Included Studies

Relevant literature was found from 15 countries (Table 1). The greatest number of studies took place in the UK (25.5%) and USA (17.0%), followed by Australia (10.6%) and South Africa (10.6%). Other countries included Japan (6.3%), China (4.2%), Brazil (4.2%), Korea (4.2%), Germany (4.2%) and several others each contributing 2.2% of the total evidence base.

**Table 1:** Geography of the included literature

Country	Number of studies	Percentage of studies (%)
UK	12	25.5%
USA	8	17.0%
Australia	5	10.6%
South Africa	5	10.6%
Japan	3	6.3%
China	2	4.2%
Brazil	2	4.2%
Korea	2	4.2%
Germany	2	4.2%
Canada, France, Portugal, Switzerland, Ireland, Italy	1 each	2.2% each
All	47	100%

#### Study Design

**Fig. 2:**
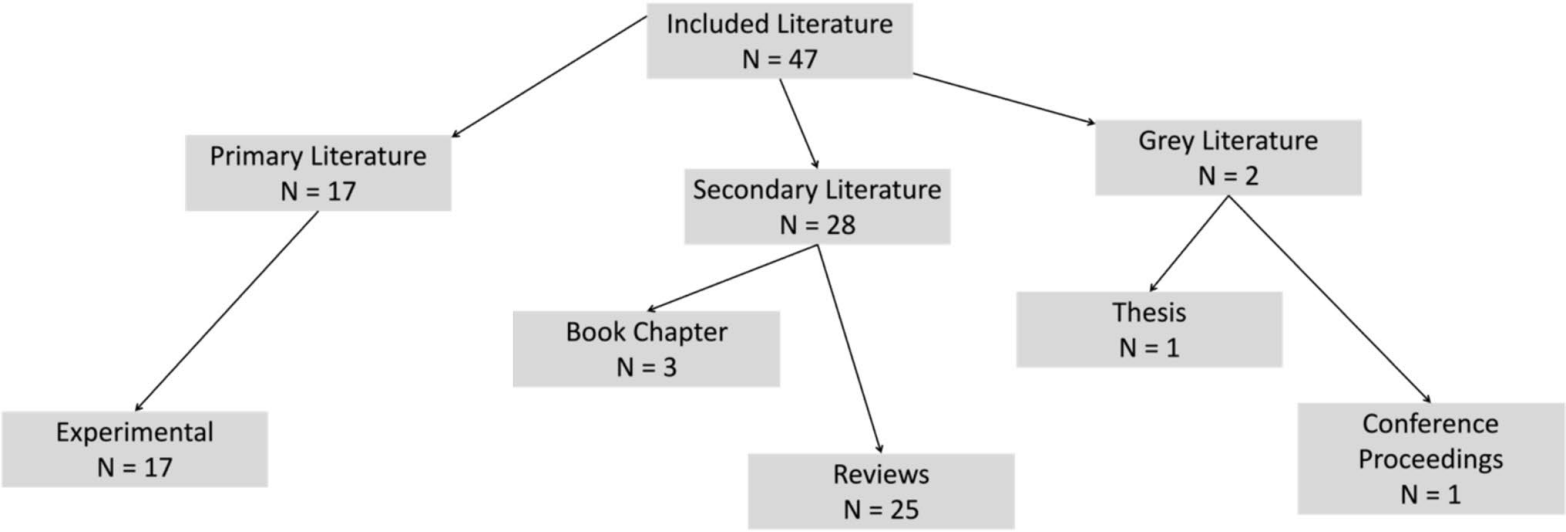
Taxonomy of research designs in the included literature

The research included in this scoping review varied pertaining to the study design, sample populations and aims. The formal quality assessment of each included article was not undertaken, which is consistent with the purpose of a scoping review—to provide a narrative or descriptive overview of the available evidence base on a given topic [23]. Of the 47 included pieces of literature, 17 (36.1%) were considered as primary research, 28 (59.6%) secondary literature and 2 (4.3%) considered as grey literature. A taxonomy of the included research is shown in Fig. 2. For the primary literature, all studies were of experimental design, which mainly reported the effect of a recovery intervention on the restoration of some measure of athletic performance, or the reduction of fatigue induced by competition or training. The secondary literature consisted of systematic or narrative reviews (*n* = 25), and three relevant book chapters were also included. The grey literature consisted of one thesis chapter and one conference proceedings. These sources of literature primarily focused on general recovery recommendations based upon guidelines from other sports, given the lack of golf-specific studies to review.

#### Theme of the Scoping Review

**Fig. 3:**
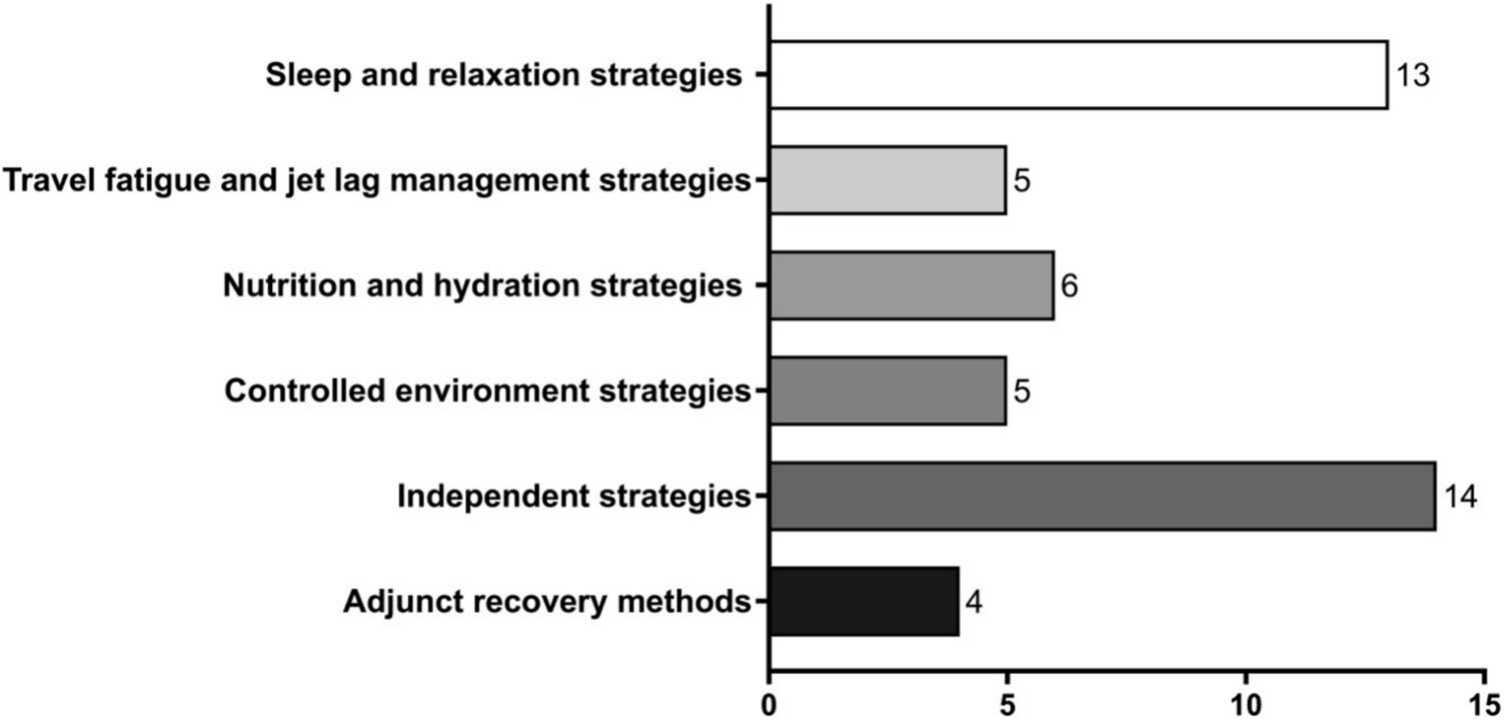
Main themes emerging from this scoping review relating to recovery strategies for golfers

As a consequence of our findings, the literature was categorised into six common themes (Fig. 3):

1. Sleep and relaxation strategies for golf recovery (*n* = 13).
2. Travel fatigue and jet lag management strategies for golf recovery (*n* = 5).
3. Nutrition and hydration strategies for golf recovery (*n* = 6).
4. Controlled environment strategies for golf recovery (*n* = 5). Note, these primarily included methods such as massage, cryotherapy, ice baths.
5. Independent strategies for golf recovery (*n* = 14). Note, these primarily included methods that do not rely on being in a laboratory or expensive equipment (e.g. mobility, flexibility, compression garments, cognitive recovery methods).
6. Adjunct recovery methods (*n* = 4). Note, these primarily included methods that have not been researched in detail (e.g. photo-biomodulation, infra-red, saunas, hyperbaric chambers).

## Thematic Overview

### Sleep

Sleep should be one of the cornerstones of any athlete’s recovery and performance strategy. This sub-section will provide a brief overview relating to how sleep influences both physical and cognitive performance. Melatonin is a hormone produced by the pineal gland within the brain, with the onset of secretion occurring approximately 2 h prior to habitual bedtime (termed dim light melatonin onset) and aligning with the start of the biological night [25]. Its production follows a circadian rhythm, which in turn, is regulated by a central “master clock”, the suprachiasmatic nucleus, as well as peripheral clocks located in most cells of the body. The master and peripheral clocks of the circadian system synchronise through *zeitgebers* or time-givers. The most common time-givers are: sleep–wake transition, physical activity, social cues, meals and light, with the latter regarded as the most influential given that photic stimuli (via the retino-hypothalamic pathway), stimulates or inhibits the suprachiasmatic nucleus [25]. For example, exposure to light, especially blue light from screens, can inhibit melatonin production, which is why reducing screen time and dimming the lights before bed is often recommended to promote better sleep quality, as is ensuring the room is as dark as possible. Additionally, our body’s circadian rhythm naturally causes a drop in core body temperature throughout the evening (reaching a nadir at approximately 04:00 depending on chronotype), further stimulating sleep as well as the maintenance of it. This is why athletes are often advised to keep their bedroom cool at night and some also advise taking a warm bath before bed, with the latter facilitating the natural decline in core temperature that occurs before sleep. This cooling process signals to the brain that it is time to prepare for sleep, promoting relaxation and drowsiness.

Sleep is crucial for physical and cognitive performance, as well as recovery and adaptation. It is often labelled the “big rock” of recovery as its positive effects are significant [26], yet athletes (and golfers in particular) face challenges in optimising sleep. As such, educating players and providing guidelines for sleep hygiene is fundamental practice by performance and coaching staff. By way of example, in 146 Brazilian Olympic athletes preparing for the Rio 2016 Games, it was found that 36% had a sleep disorder, and 53% of athletes reported sleep complaints, with the most prevalent being insufficient sleep or waking up tired (32%), followed by snoring (21%), and insomnia (19.2%) [27].

With respect to physical performance and recovery, sleep allows for muscle growth and repair (as well as bone and body fat mass regulation), facilitated principally by the release of growth hormone occurring during the early stages of sleep [28]. Furthermore, there is a down-regulation of the hypothalamic–pituitary–adrenal axis and the sympathetic nervous system and, thus decreases in plasma cortisol, epinephrine and norepinephrine levels [29]. Sleep also restores energy stores by replenishing glycogen and strengthens the immune system by producing cytokines that help fight infection and inflammation [30]. Equally, good sleep improves coordination and reaction times, and reduces the perception of effort, meaning athletes can train with greater intensity. For example, following approximately 30 h of acute sleep deprivation in team-sport athletes, significant decrements in performance were noted including: reduced repeat sprint ability, muscle glycogen content, peak voluntary force and activation, and negative perceptual strain [31].

From a cognitive standpoint, sleep plays a key role in memory consolidation by transferring short-term memories to long-term storage during rapid eye movement sleep [32]. It enhances learning and problem-solving abilities, assists with concentration and decision making, and also helps regulate emotions and reduces stress [33]. During sleep, particularly in rapid eye movement stages, the brain processes emotional experiences, helping to reduce the emotional intensity of those experiences and improve mood regulation. Conversely, a lack of sleep can lead to increased levels of stress hormones such as cortisol, resulting in irritability and fluctuations in mood. Sleep is also essential for regulating hormones that control hunger and appetite [34]. During sleep, the body balances the levels of ghrelin and leptin, hormones that signal hunger and fullness, respectively. Conversely, a lack of sleep increases ghrelin levels and decreases leptin levels, leading to increased hunger (and potentially calorie intake). Poor sleep can also lead to increased blood pressure, higher levels of stress hormones and inflammation, all of which are risk factors for various cardiometabolic diseases.

Naturally, obtaining optimal and good-quality sleep for any athlete is essential to help optimise recovery. However, owing to the changing time zones and accompanying jet lag (discussed in the next section), this is a considerable challenge for professional and elite amateur golfers. Regardless, to improve both sleep onset and quality, below are some guidelines for practicing good sleep hygiene [26]. Of note, some of the suggestions below will only be achievable for golfers when not travelling and/or are based in one location for an extended period of time.

- **Maintain a consistent sleep schedule:** Going to bed and waking up at a similar time every day supports the regulation of the body’s internal clock, making it easier to fall asleep and wake up naturally. This can also reduce cortisol levels and muscle catabolism, which may potentially enhance performance [35].
- **Create a relaxing bedtime routine:** Identifying individualised stress-reducing and calming activities such as reading, taking a warm bath or practicing relaxation exercises (e.g. meditation or deep breathing exercises) provides a signal to the body that it is time to relax, helping a more natural transition into sleep [36].
- **Bedroom temperature:** Keep the bedroom cool, ideally between 60 and 67 °F (15–19 °C). A cooler room temperature helps facilitate the natural drop in core body temperature that occurs during sleep and as part of our circadian rhythm [37].
- **Ensure darkness in the bedroom**: Use blackout curtains or an eye mask to create a dark sleep environment. Darkness signals to the brain that it is night time and helps regulate the production of melatonin, the hormone that promotes sleep [38]. Light exposure, especially blue lights from electronic devices and artificial sources, suppresses melatonin production and delays the onset of sleepiness.
- **Watch the diet:** Avoiding heavy meals, caffeine and alcohol close to bedtime prevents disruptions in sleep caused by indigestion or the stimulant effects of caffeine and alcohol. Caffeine, in particular, can delay the release of melatonin, and block adenosine receptors, thus temporarily promoting alertness, making it harder to both fall asleep and stay asleep [39]. Given caffeine has a half-life that varies between 2.7 and 9.9 h, ideally individualised advice should be offered. However, generic guidance would be to avoid caffeine after 14:00 unless used as a performance enhancement strategy during competition [40].
- **Stay active:** Regular physical activity promotes better sleep by reducing stress and tiring the body, resulting in the accumulation of adenosine molecules. However, avoiding vigorous exercise close to bedtime limits stimulation of the sympathetic nervous system and thus, increased concentrations of adrenaline and noradrenaline. These “fight or flight” hormones increase heart rate, blood pressure and alertness, which are counterproductive to the relaxation needed for sleep [41].

### Travel Fatigue and Jet Lag Management for Golf Recovery

As highlighted in Sect. 1, professional golf tours have schedules that include tournaments on multiple continents, with frequent transitions between time zones [2]. Naturally, this has an interrupting effect on critical components of recovery, such as consistent sleep patterns [26]. The combined demands of travel and regular time zone changes (i.e. travel fatigue and jet lag) can challenge physical function and cognitive recovery in golfers. Athletes participating in sports with less frequent travel can employ pre-adaptation, phase-advance or phase-delay strategies, depending on their direction of travel [2, 25, 42–44], some of which can be used by golfers for priority competitions. Such strategies include adjusting the time an athlete goes to bed or wakes up 3–4 days before travel, altering their light exposure throughout the day, as well as manipulating the timing of their meals [2, 43]. However, golfers can only employ some of these strategies consistently, owing to weekly competition schedules being in a variety of different locations globally. As a result of these frequent changes in location and time zones, we recommend that players invest in a “toolbox” of simple and ‘easy to adhere to’ travel strategies that can be employed immediately post-competition on a Sunday evening (assuming they make the cut) to help them prepare for the next event. Whilst an individualized approach is always preferable, we have built on the existing literature to describe a series of practical strategies broken down into graded steps spread across the different stages of travel, that golfers will experience.

#### Step 1: Distinguish Between Travel Fatigue and Jet Lag

Travel fatigue is defined as temporary travel-related fatigue or exhaustion and can follow any long journey, including: car, bus and train trips even without crossing time zones. It follows a period of prolonged inactivity, with potentially irregular sleep, restricted food choices, dehydration and other factors associated with long-distance travel [2, 42, 43]. Jet lag may co-exist with travel fatigue but follows rapid long-distance travel, crossing three or more time zones. It is caused by rapid trans-meridian travel and the body clock becoming discordant with the time zone at the new destination [2, 25, 42, 43].

#### Step 2: Immediately Post-competition

In the immediate post-competition phase the focus should be on acute recovery for golfers. The energy cost of a round of golf during competition can exceed 1000 kilo-calories [45], although the exact value will be specific to the individual and the topography of the course. In addition to this energy expenditure, there may be some glycogen depletion, muscle damage and dehydration [46]. To address these needs, golfers are advised to repair potentially damaged muscle with high-quality protein, replenish fuel stores with appropriate amounts of carbohydrates, and rehydrate with fluids and electrolytes [46, 47]. Should players have more time, they can complement this with modalities such as cognitive recovery, hands-on massage, compression garments and general relaxation, followed by an additional recovery meal and good quality and quantity of sleep if possible [25, 43]. Finally, when playing multiple tournaments in a row, players are encouraged to plan as much as possible in advance, to reduce unnecessary stress and anxiety. Some suggestions from Dunne et al. [2] include:

- Ensure documentation is in order (e.g. passport and visa if required).
- Take the most direct route to the next destination, with the least number of stopovers.
- Get enough sleep and rest before the journey.
- Prevent illness, by developing consistent good habits (e.g. enough rest and sleep, well-balanced nutrition and hydration, use of antibacterial hand gel).

#### Step 3: At the Airport

Attention for golfers at the airport should now shift towards preparing for travel, including purchasing some airport essentials. Following check-in and proceeding through airport security, players should purchase antibacterial hand gel, if they do not have any already, for regular use throughout the trip. Depending on the duration of travel, golfers should also consider purchasing some travel meals, snacks and water, as the food service provided by airlines can sometimes be unreliable, and the in-flight service also has the potential to disturb sleep.

#### Step 4: During Travel

First, during travel golfers may wish to change the time zones on their devices (e.g. phone or watch) to that of the new destination. To support quality sleep, they are advised to align their sleep with the place of departure as their psychological drive for this rest is higher, making sleep easier to initiate. Should golfers struggle to sleep consistently, then a ‘sleep-when-possible’ approach is advised during travel. Added considerations include: (i) good-quality ear plugs; (ii) an eye mask to block out light; and (iii) recovery leggings or flight socks to aid venous return and decrease the risk of deep vein thrombosis. If golfers purchase food in the airport to consume during travel, then an in-flight meal and snack timings may be adjusted according to the destination time zone [42–44]. Food choices should remain familiar with new foods being avoided where possible. In addition to planning their meal timing, golfers are advised to follow a pattern of eating regular small meals to meet their energy requirements and avoid deficiencies. Consuming regular but smaller meals containing sufficient protein, micronutrients and fibre (e.g. fresh or dried fruit), pre-prepared by the athlete or their team, will aid recovery and support immune function [2].

Throughout the flight, golfers should get as much rest and sleep as possible. They are advised to drink regularly and follow a hydration plan that was configured pre-travel. Regular sips of non-alcoholic and non-carbonated drinks are best, with priority given to water with electrolytes [47]. Naturally, alcohol and caffeine should be avoided or limited. The recycled air of the aircraft makes it easier to lose moisture during breathing, which has the capacity to increase dehydration, which in turn, can increase the risk of contracting an illness [2, 25, 42–44]. Needing to visit the bathroom relatively regularly throughout the flight is a useful practical indicator that the player is drinking enough. In addition to staying hydrated, chewing gum can also aid the production of salivary immunoglobulin A and reduce the risk of infection [2]. Finally, when awake, stretching and, if possible, doing some movement-related exercises up and down the aisle is advised to retain sufficient joint mobility.

#### Step 5: Arrival at New Destination

Having arrived at the new destination, the full focus is now on recovering from the flight and preparing for the competition likely starting on Thursday. Given the need for golfers to be able to get into the desired swing positions, some level of mobility work and foam rolling is advised within the first 24 h of landing to help restore range of motion and reduce joint stiffness [48]. In addition, it is suggested that relevant mobility work is repeated as part of a routine warm-up, prior to practice and competition, as some effects are unlikely to last beyond 10–15 min [49]. Finally, heavy or intense resistance training should be adapted if golfers feel fatigued or sluggish.

### Nutrition and Hydration Strategies for Golf Recovery

Prior literature has undertaken a more in-depth analysis of the nutrition and hydration requirements for professional and elite amateur golfers [46, 47, 50–53], and readers are directed to these for a more in-depth overview, specific to nutrition and hydration alone. Naturally, good-quality nutrition and hydration habits are essential to help any athlete optimise their recovery after training and competition [54–56]. When considering requirements specific to golf, tournaments are often played in a hot environment, for an extended period of time; thus, we can break recovery strategies down into three key timepoints each day: (i) post-competition; (ii) evening; and (iii) pre-bed. As a golfer comes off the course, it is suggested that they focus on the three Rs of recovery: (i) rehydrate; (ii) repair (damaged tissue with high-quality protein); and (iii) restore (depleted energy stores with appropriate carbohydrates).

#### Post-round

- **Rehydrate:** golfers should aim to consume 1.5 L of water for every 1 kg of weight lost during a round [57]. Additionally, players should also aim to include some electrolytes post-round to ensure salt that is lost through sweat is replaced, although this does not necessarily require specific drinks given that these electrolytes are also readily available in many foods commonly consumed post-exercise.
- **Repair:** golfers should consume at least 20–40 g of high-quality protein soon after a round. This can be achieved by having meat-, fish- or plant-based protein foods available in the players’ lounge as part of a post-round meal. If a player has many media responsibilities or wants to go straight back to practice, nuts, milk, Greek yoghurts or dairy-based smoothies are also viable options. Adding two to three servings of fruit and/or vegetables alongside the aforementioned dairy options post-round also supplies some additional antioxidants that may further aid repair [46].
- **Restore:** restoring depleted fuel stores can be achieved by having some high-quality carbohydrates in a post-round meal, such as pasta, potatoes or rice. In addition, adding some fruit will add additional fructose to the meal, which has been suggested to be important in the replacement of liver glycogen [58].

#### Evening and Pre-bed

For evening meals, the focus is comparable to previous suggestions. Adequate hydration in the evening helps players continually replace the fluids lost during the day and puts them in a better position to wake up hydrated for competition the following day. Ensuring a regular supply of quality protein ensures the muscles have the raw materials required to continue to repair themselves. Finally, including carbohydrates in the evening meal, will help replenish depleted muscle and/or liver glycogen stores. Pre-bed, the focus should be on delivering some high-quality protein to aid overnight recovery given that pre-bed ingestion of protein has been shown to increase both mitochondrial and myofibrillar muscle protein synthesis [59]. With this in mind, one option for golfers is Greek yoghurt, which should be easily accessible, regardless of which country they are competing in, helping to promote a ‘food first’ approach to sport nutrition [60].

### Controlled Environment Strategies for Golf Recovery

#### Massage

While there are various types of massage, very few studies have been conducted specifically in golf. Lim [61] recruited 20 golfers of varying experience levels to use a massage chair for 20 min, focusing on treatment around the gluteal, lower and upper back muscles. The results showed significant improvements (*p* ≤ 0.03) in clubhead speed (CHS), ball speed, carry and total distance. Despite these positive findings, it seems conceivable that improvements in tissue extensibility were a result of improvements in perceived recovery or increases in tissue temperature. It is important to note, though, that tissue temperature was not measured; however, this seems like a plausible explanation when the duration of the intervention was 20 min. Thus, a well-designed warm-up routine may offer comparable benefits [12]. In support of this, Quinn et al. [62] undertook a comparison of trigger point therapy in the hip flexor muscle with medicine ball exercises versus trigger point therapy (again in the hip flexor) with stretching of the same muscle group on CHS, ball speed, distance and smash factor in 100 male golfers. Although raw data were not reported, the group that included medicine ball throws significantly improved their backswing hip turn (*p* = 0.02) and accuracy (*p* = 0.02) relative to the control group. To the authors’ knowledge, this represents all the information pertaining to empirical studies investigating the effects of massage on golfers. However, some broader benefits may also exist, which should be acknowledged based on the wider literature. First, massage may provide some acute improvements in range of motion and delayed-onset muscle soreness (DOMS) [63]. Additionally, the mechanical pressure on muscles may induce changes in parasympathetic activity (e.g. heart rate, blood pressure, heart rate variability) and hormonal levels (e.g. cortisol) that enhance a player’s ability to relax post-competition [63]. Moreover, including massage as a part of regular recovery regimens may reduce an athlete’s anxiety and improve their mood and wider cognitive state, which can additionally optimise recovery [63]. Thus, despite the limited amount of empirical investigations conducted in golf specifically, massage may still have its place in the over-arching recovery process for golfers, especially in areas that are prone to injuries such as the lower back, wrist, elbow and neck [64, 65]. Specifically, for players who feel stiff or sluggish, massage may be a useful strategy prior to starting a warm-up routine, given that previous research has shown no detrimental effect on subsequent performance (albeit in the sport of boxing) [66].

#### Cold Therapies Including Cold Plunges and Ice Baths

Cold therapies (or cryotherapy) is a term that can be used to describe numerous treatments that focus on the application of ‘cold’ to elicit therapeutic benefits [67]. By facilitating heat dissipation, cryotherapy can reduce tissue and core temperatures to mitigate thermal strain [68]. Concurrently, it can also alter muscle blood flow to reduce inflammatory responses and secondary tissue damage [69], and provide analgesic effects through decreased neural conductivity, enhancing perceptual recovery [70, 71]. The use of cryotherapy for athletic recovery has been steadily increasing over the last 70 years, and in the last decade, the development of cold plunges, whole-body cryotherapy chambers, portable cryo-pneumatic systems and phase change materials has meant that practitioners and athletes have numerous options to choose from [72].

Outdoor sports such as golf require athletes to contend with fluctuations in the natural environment that could impact on their performance. Given that an 18-hole round is expected to take 5–6 h, and that tournaments are usually conducted over several consecutive days, golfers can be exposed to moderate or high thermal loads [73], which can potentially reduce motor-cognitive performance or increase central nervous system fatigue [74]. For example, during the FedEx St. Jude Championship playoffs held in Memphis, Tennessee in August 2023, temperatures reached 43.9 °C (111 °F) [75], with similar conditions experienced at the following tournament in Atlanta. Cold therapy strategies can be used both during and immediately post-round to mitigate the negative impacts of excess heat storage on performance and subsequent recovery. During rounds, cold towels around the neck or even ice vests could be an option when players are waiting to play their next shot (noting that wait times can range between 5 and 15 min), to reduce heat storage and skin temperature [76]. During play, cooling during very hot and humid conditions can be further enhanced by replacing traditional ice packs with an alternative phase-change material with a set point of 15 °C, which can safely provide > 3 h of constant cooling, by seeking shade, and ingesting cold fluids [77].

An 18-hole round of golf in a professional competitive setting is considered moderate-level aerobic physical activity [45]; however, average energy expenditure data do not consider dynamic explosive movements such as those typified by a golf swing. Although research into exercise-induced muscle damage is notably sparse in golf, evidence suggests that an 18-hole round of golf can elicit increases in inflammation, and result in structural muscle damage in healthy young men [78]. Although Yasuoka et al. [78] did not recruit golfers, which may have impacted upon the magnitude of damage experienced by participants, an earlier study from Suh et al. [79] reported that creatine kinase (CK) was elevated in golfers for at least 24 h following a practice session, irrespective of the competitive level, with the extent of biochemical disturbance increasing as practice volume increased (100, 200 or 300 shots). However, it is important to note that CK levels are likely to remain elevated for longer durations in team sport athletes [80]. Taken together, these studies indicate that golf performance, either during practice sessions or in competitive scenarios, may result in mild inflammation and structural muscle damage that could impact upon subsequent performance in competitions [78]. Whilst this certainly warrants further investigation, it would be reasonable to suggest that implementing post-exercise cold therapy (e.g. cold plunge, “ice bath” or whole-body cryotherapy) could help to attenuate increases in interleukin-6 and CK in golfers following training and competition, thus expediting recovery. This is most likely to be beneficial when the golfer is undertaking unaccustomed exercise (e.g. more than 18 holes in a day), in hot and humid conditions, or when a golfer anticipates muscle soreness. Outside of these circumstances, muscle damage, and benefits from a cold plunge or ice bath may be minimal unless the player exhibits a strong preference or receives cognitive benefits.

A recent systematic review with meta-analysis and meta-regression concluded that cold-water immersion (CWI) was more effective than active recovery, contrast water therapy and warm-water immersion for the reduction of muscle soreness post-exercise [81]. Cold-water immersion protocols vary by temperature and duration (usually between 4 and 15 °C for between 4 and 15 min), though specific procedures may be partially governed by athlete tolerance to cold, or the desire to target specific symptoms. Colder temperatures result in a greater initial ‘cold shock’, which can increase athlete discomfort during immersion [82]. Therefore, longer durations at slightly warmer temperatures may be preferable for some. However, Moore et al. [81] demonstrated that there was a dose–response effect of lower temperatures and shorter durations positively influencing recovery of muscle power compared with active recovery at 24 h post-exercise [81]. In addition, and to provide some more specific guidance, Vromans et al. [83] examined the dose–response relationship between CWI and its effects on decreasing muscle tissue temperature. The evidence suggests that the most optimal dose was when the body was fully immersed with a measurement depth of 300 mm (*r* = 0.87). Further to this, significant reductions in tissue temperature (and therefore inflammation) can be achieved with immersion for 11 min with a 10 °C water temperature. Furthermore, and important to golf, CWI and cryotherapies may blunt some of the adaptation effects from strength and conditioning training within the first 4 h [84], which is an important consideration for players who prioritise increasing CHS, ball speed and distance [85]. In light of this information, we provide the following practical recommendations for golfers:

- When exposed to high ambient temperatures and/or humid conditions, consider pre-competition cooling strategies (e.g. utilising cold towels) and even between shots, or during breaks in practice, to attenuate excess heat storage.
- After completion of a round (particularly in conditions of high thermal load, major competition or unaccustomed exercise), golfers should consider using CWI (ensuring submersion of the upper limbs) to alleviate DOMS and inflammation.
- During training or non-priority competition weeks, players may be advised to avoid CWI or cryotherapy to maximise potential adaptation from resistance training.
- Water temperatures of approximately 10 °C and immersion durations of 11 min, respectively, are likely to be effective for optimising post-exercise recovery from golf, although golfers may exhibit strong individual preferences.

### Independent Strategies for Golf Recovery

#### Mobility and Flexibility

When considering mobility and flexibility as a recovery modality, the first area to consider is stretching [86–89]. Specifically, the purpose of static stretching is to acutely move a limb to its end range of motion for 15–60 s, so that tissue length can increase [90]. Current evidence indicates that from a force production, muscle power and jump height standpoint, static stretching predominantly has a detrimental effect [90]. In contrast, results show that static stretching typically has a positive effect on increasing range of motion and reducing passive stiffness of muscles [91]. Thus, if a golfer is lacking in mobility of specific areas (e.g. hips or thoracic spine), static stretching may be a viable option to assist with reducing stiffness and discomfort, which in turn, may have in part been caused by insufficient range of motion in the first instance. As a further point of consideration, however, static stretching does create mechanical tension [77], which in turn, has the ability to further damage tissue. Thus, if and when applying static stretching, the suggestion is to ensure that players hold positions within a ‘comfortable degree of stretch’ to avoid detrimental effects on recovery. However, it should be acknowledged that recent evidence has indicated that there is limited association between measures of flexibility and CHS [13]. Therefore, golfers and practitioners should not consider improving flexibility as a direct means of improving golf performance; rather, as a small piece of the recovery process in certain instances.

A second area for golfers to consider is foam rolling. From an athletic performance standpoint, current evidence indicates no meaningful association between foam rolling and measures of strength, jump height and sprint performance [48]. In contrast, foam rolling can demonstrate significant improvements in flexibility and a perceived reduction in pain [48]. Thus, and similar to static stretching, foam rolling may be a useful activity as part of a golfer’s wider recovery strategy. However, it is worth recognising that in the authors’ experience, we have often seen athletes foam roll, moving back and forth over muscles at a speed that is likely unconducive to sustainable improvements in range of motion or reductions in tissue stiffness. Our suggestion is that golfers (and all athletes) should ensure they move very slowly over the tissue in question. Furthermore, some muscles (e.g. piriformis and tensor fascia latae) may be better served by resting on tender points within the tissue and remaining stationary until a reduction in pain is perceived, as opposed to rolling back and forth over the tissue, which is likely to be less effective.

Handheld percussive massage devices (i.e. massage guns) are intended to reduce muscle soreness by delivering targeted vibration to soft tissues. Although popular with some athletes, evidence of efficacy is limited and caution is recommended when using massage guns immediately after strenuous lower body exercise [92]. The conclusion from a recent systematic review was that: *“massage guns can help to improve short-term range of motion, flexibility and recovery-related outcomes, but their use in strength, balance, acceleration, agility and explosive activities is not recommended”* and that in some cases, they may exhibit deleterious effects [93]. In addition, when interpreting these findings, it appears that these devices have been used prior to exercise; thus, some further investigations are warranted to determine their efficacy as a recovery modality, when inflammation and muscle damage are likely to be more prevalent.

#### Compression Garments

Exercise containing eccentric contractions will elicit greater tissue damage than concentric alone [94], and the symptoms can result in a number of detrimental effects on measures of athletic performance, such as reductions in strength, rate of force development and reduced range of motion [95]. Prior research in golf has repeatedly outlined the importance of physical attributes such as maximal and explosive force production [1, 13, 96, 97]. Thus, reductions in these capacities could have a negative impact on key proxy measures such as CHS, which in turn, may also impact other performance measures in golf such as ball speed, distance and even strokes gained [16]. However, although previous research has shown that wearing a compression top slightly restricts a golfer’s trunk rotation during the swing, with a marginal increase in CHS, neither of these changes exhibited statistical significance [98]. Thus, from the limited body of evidence in golf, compression garments may be a preference for players, but some caution should be exercised when considering the magnitude and quality of evidence. Despite this, some additional (logic-led) factors can be considered from the wider research. For example, and when focused on compression garment literature more generally, a meta-analysis undertaken by Hill et al. [94], showed that compression garments exhibited significant beneficial effects for DOMS (*g* = 0.403), muscle strength (*g* = 0.462), muscle power (*g* = 0.487) and CK (*g* = 0.439), which can be used as a marker to signify muscle damage. Any post-exercise benefit from compression garments will undoubtedly be related to the type, duration and intensity of exercise [94], noting potential benefits regarding recovery from travel. Whilst the exercise intensity of golf is lower than many other sports, players do compete for 2–4 consecutive days, often for multiple weeks in a row. Thus, and as mentioned in Sect.1, the cumulative effect of force being approximately seven times body mass during the swing, which may be repeated circa 2000 times in a given week and an average of 40–80 km walked per week (which are likely to be repeated for multiple weeks in a row), will unquestionably take a cumulative toll on the body.

#### Cognitive Recovery

At the professional and elite amateur levels, golf is a cognitively demanding sport owing to the requirement for sustained concentration, with typically > 400 h of competition throughout the course of a year. In support of this, golfers have themselves previously described the cognitive challenges of travel, long days of practice, and > 150 days away from friends and family per year [99]. Further to this, many have reported on the financial pressures they face, and the challenges of being in an environment where friends are generally also competitors [100]. Symptoms of performance anxiety, erratic moods and loneliness are relatively prevalent in professional and elite amateur golfers, and proactive cognitive recovery should be prioritised to maximise subsequent performance, and mental health [99, 101, 102].

In previous sections, this review has outlined the cognitive benefits of good sleep hygiene, and in particular, gaining adequate quality and quantity of sleep. This can be achieved during night-time hours employing strategies previously described, while also napping for 20–60 min can be a useful adjunct to night-time sleep. Down time and relaxation are also an important component of athletic recovery [103], while many players enjoy maintaining social connections with those at events, and prioritising connecting with family and friends at home. Purposeful relaxation and psychological detachment has been described in professional tennis players, who share similar travel and scheduling demands [104], and efforts to factor in downtime during weeks, as well as having some time away from the profession each year may help sustain high performance in athletes [103]. Various psychological relaxation techniques are described to enhance recovery in sports, including golf. These may include: meditation, breathing techniques, music, and more recently, virtual reality [105]. Consistent with this, infrastructure and support to promote cognitive health and recovery have been included in some professional golf events, such as dedicated relaxation areas, virtual reality, areas with support for meditation, breathing techniques, reading and journaling, sleep pods, and the inclusion of mental health and well-being professionals [106]. Some golfers also describe relaxation and cognitive benefit from massage, and cold plunges/ice baths, which are also frequently provided at competition venues.

### Adjunct Recovery Methods

Recent years have seen a proliferation of other adjunct therapies advocated for recovery, with variable evidence of efficacy, cost and ease of implementation. Each athlete may have personal preferences, and in some cases, commercial considerations. We briefly discuss some specific methods below, noting that there are many others with very limited empirical evidence supporting their use in practice.

#### Photo-Biomodulation and Infra-Red

Near-infrared (NIR) phototherapy is a non-invasive light treatment which has shown some promise in supporting muscle recovery. Despite the body of evidence supporting the efficacy of NIR light therapy, the mechanistic underpinnings have not yet been fully elucidated. Findings from ex vivo research utilising C_2_C_12_ murine skeletal muscle cells demonstrated that NIR light exposure induced adaptations associated with mitochondrial biogenesis, which could contribute to the reported therapeutic benefits of NIR phototherapy [107]. Peng et al. [108] conducted a meta-analysis which suggested that NIR phototherapy can help protect against strength loss (as measured by muscle torque) and reduce biochemical markers of muscle damage, such as CK and DOMS. The effects are most pronounced within the first 24 h post-exercise, making timing a crucial factor for maximising recovery benefits. This protective effect on muscle strength and soreness suggests that NIR phototherapy could be valuable for golfers, particularly during intense training or tournaments with limited recovery time. Additionally, research on other athletes has found that pre-exercise NIR may increase endurance and reduce fatigue markers [109], which could offer performance benefits for golfers who face long practice sessions or competitive rounds. Finally, practitioners and golf coaches should also be aware of the dose-dependent nature of NIR phototherapy, where optimal results were observed using specific energy and power levels (e.g. approximately 24 J/cm^2^ and 5495 mW/cm^2^ for energy and power density, respectively). Collectively, the body of evidence supporting this method is somewhat limited and the ability to implement it during tournament weeks is also challenging, inhibiting its overall viability as a recovery method.

#### Heat Therapies

While limited research specifically examines heat therapy in high-level golfers, evidence from other sports suggests that heat treatments may aid recovery by promoting muscle relaxation and reducing soreness [81]. Evidence from animal studies provides some insight regarding the mechanistic underpinnings of heat application for recovery following muscle damage or injury. Studies utilising hot water immersion, thermal blankets or environmental chambers to apply heat report a greater size and number of muscle fibres [110, 111], increased heat shock proteins (HSP72) [111, 112], faster macrophage infiltration [110], increased expression of growth factors [110], increased proliferation of Pax7+ satellite cells [110, 111] and increased recovery of muscle mass relative to body mass [111, 113] during re-loading compared with the control condition. However, Skorski et al. [114] found that spending time in a sauna post-exercise can hinder recovery if followed by high-intensity activity too quickly thereafter. This effect suggests that heat stress may elevate core temperature, train the cardiovascular and thermoregulatory systems, and potentially increase fatigue [114]. Similarly, Peterson et al. [115] noted that while heat can enhance blood flow and muscle relaxation, it may also increase DOMS and inflammation if used immediately after exercise. With this in mind, it is suggested that golfers avoid or limit heat therapy between rounds, especially during competition weeks.

#### Hyperbaric Chambers

Hyperbaric oxygen therapy, which involves breathing oxygen in a pressurised chamber, has been studied for its potential to speed up recovery and improve performance in high-intensity sports. Mihailovic et al. [116] demonstrated that post-exercise hyperbaric oxygen therapy at modest pressure (97% O_2_; 1.3 atmospheres absolute) improved subsequent cycling performance, increased heart rate variability and reduced perceived exertion, suggesting faster cardiovascular recovery and enhanced recovery perception. However, as Mallette et al. [117] noted, the results on hyperbaric oxygen therapy exhibit considerable variation across studies, with outcomes depending on factors such as oxygen concentration and exercise intensity, which limits the ability to make definitive recommendations. It should also be noted that golf generally provides low-to-moderate aerobic exercise intensity, so the potential benefits from this method may be less evident.

### Summary

**Fig. 4:**
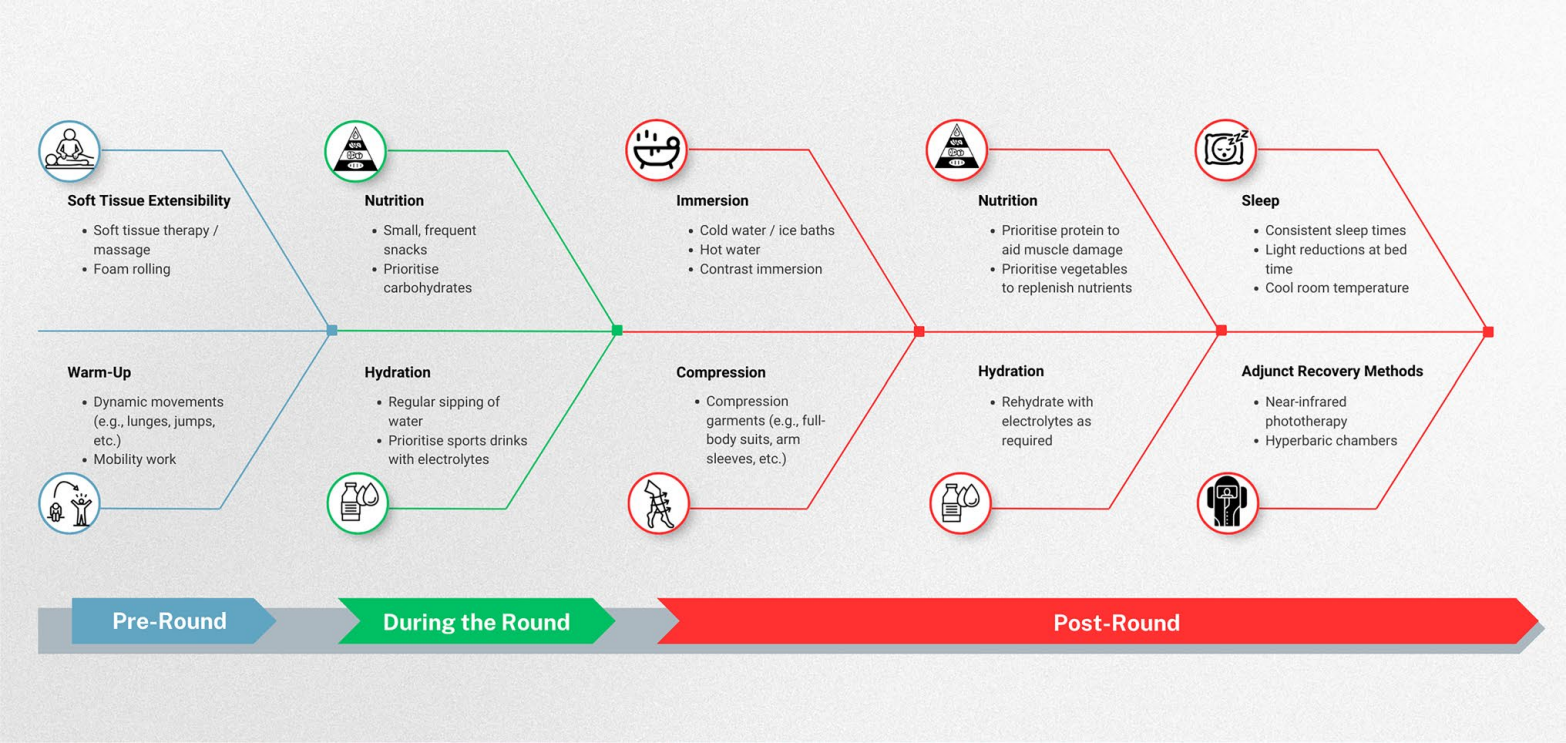
Timeline of different performance recovery **options** for golfers on a given competition day. *Note:* Recovery strategies may be specific to each player's preferences and competition environment.

**Fig. 5:**
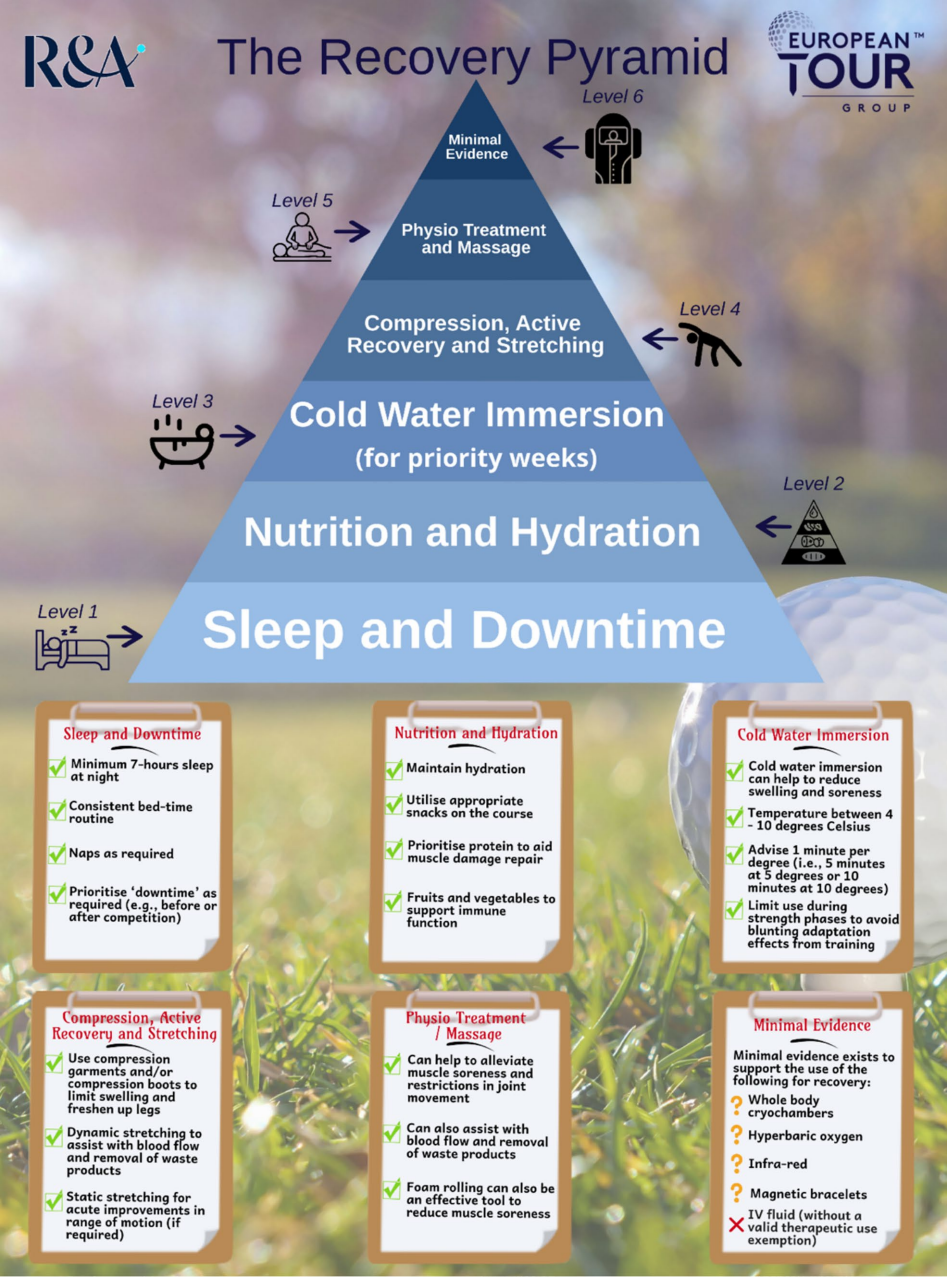
A proposed recovery pyramid for golfers. The most important factors are at the bottom, with the size of the text in the pyramid signifying importance. *IV* intravenous

Despite the volume of literature pertaining to recovery in golf being relatively sparse, there are a number of well-established practices that golfers should prioritise for their own recovery based on the best available evidence, just like any other athlete. In addition, there are some unique aspects to an elite or professional golfer’s lifestyle (e.g. week-to-week travel, changing time zones), which present some challenges requiring a more bespoke approach. To aid in the dissemination of this multi-faceted problem for golfers, we have created two summary figures. The first (Fig. 4) is a timeline of different performance recovery options that golfers and practitioners may consider at different stages of an example competition day. This has been broken down into pre-round, during the round and post-round. The second (Fig. 5) is a ‘recovery pyramid’ infographic, which offers a broader take-home message on the most impactful strategies for the golf athlete. This pyramid prioritises these recovery strategies from bottom to top, representing a strong foundation for some of the most basic principles first and foremost.

## Conclusions

The lifestyle of a professional and elite amateur golfer presents many unique challenges, some of which make meaningful recovery difficult to achieve. For many players, with time zone changes being a regular occurrence, consistent high-quality sleep can be problematic, and strategies aiming to maximise quality and quantity of sleep are a priority. Whilst there is no substitute for this, the effects these lifestyle challenges likely have on sleep quality do mean that even greater attention should be paid to other aspects of the recovery process. As such, additional strategies are likely to include: ensuring nutrition and hydration habits are as optimal as possible, relaxation and psychological recovery are prioritised, cold therapies are considered in particular environments or when recovery is the focus, and general consistent warm-up and cool-down activities are undertaken, which may include stretching, foam rolling and massage. Ultimately, if golfers want to improve their chances of consistently competing at the highest level, taking care of their over-arching physical health and well-being is now one of the most important factors helping to sustain their golf performance over time.
